# Scaling sensor metadata extraction for exposure health using LLMs

**DOI:** 10.1093/exposome/osag008

**Published:** 2026-03-13

**Authors:** Fatemeh Shah-Mohammadi, Sunho Im, Julio C Facelli, Mollie R Cummins, Ramkiran Gouripeddi

**Affiliations:** Department of Biomedical Informatics, The University of Utah, Salt Lake City, UT 84108, United State; College of Nursing, The University of Utah, Salt Lake City, UT 84108, United States; Department of Biomedical Informatics, The University of Utah, Salt Lake City, UT 84108, United State; Utah Clinical and Translational Science Institute, The University of Utah, Salt Lake City, UT 84108, United States; College of Nursing, The University of Utah, Salt Lake City, UT 84108, United States; Department of Biomedical Informatics, The University of Utah, Salt Lake City, UT 84108, United State; Utah Clinical and Translational Science Institute, The University of Utah, Salt Lake City, UT 84108, United States

**Keywords:** exposure health, sensor, metadata, GPT, information extraction

## Abstract

**Background:**

The rapid evolution and diversity of sensor technologies, coupled with inconsistencies in how sensor metadata is reported across formats and sources, present significant challenges for generating exposomes and exposure health research.

**Objective:**

Despite the development of standardized metadata schemas, the process of extracting sensor metadata from unstructured sources remains largely manual and unscalable. To address this bottleneck, we developed and evaluated a large language model (LLM)-based pipeline for automating sensor metadata extraction and harmonization from publicly available exposure health literature.

**Methods:**

Using GPT-4 in a zero-shot setting, we constructed a pipeline that parses full-text PDFs to extract metadata and harmonizes output into structured formats.

**Results:**

Our automated pipeline achieved substantial efficiency gains in completing extractions much faster than manual review and demonstrated strong performance with 88.0% accuracy, 88.0% precision, 93.0% recall, and an F1-score of 90.0%.

**Conclusions:**

This study demonstrates the feasibility and scalability of leveraging LLMs to automate sensor metadata extraction for exposure health, reducing manual burden while enhancing metadata completeness and consistency. Our findings support the integration of LLM-driven pipelines into exposure health informatics platforms.

## Introduction

Sensors, defined by the NIH as tools that detect, transmit, or report data on biological, chemical, or physical processes, are characterized by metadata describing their principles, accuracy, calibration, and deployment features.^1-7^ The diversity and rapid evolution of sensors^8^^,^^9^ coupled with the diverse formats and inconsistent structures of sensor metadata create significant challenges for selecting, integrating, and utilizing optimal sensors for exposure health studies. There is a widely recognized need for systematic approaches to improve the harmonization and integration of sensor metadata, for the effective use of emerging technologies in exposure health research.^10-14^

Despite this expansion, significant challenges persist in applying sensors and sensor data to exposome research.^1^ For prospective sensor use in the quantification of the exposome, these challenges include selecting appropriate sensors, deploying and monitoring sensor networks, managing data streams, pre-processing sensor data, and integrating with other data sources for analysis.^15^^,^^16^ For secondary use of existing sensor-based measurements, including real-world data resources, for the assimilating exposomes, there is a need to find and understand information about sensor characteristics and capabilities for appropriate harmonization and utilization of data for generating exposomes.^15^^,^^16^ Generally underdeveloped data standards in exposure health, and the specific lack of standardization among sensors and sensor data, further complicate the process of sensor-based exposure health. All of these challenges become more daunting as research questions necessitate multiple and varied sensors, or measurements of varied provenance.

To address this complexity, standardized models are needed to ensure metadata is consistent and computable. The Sensors and Metadata for Analytics and Research in Exposure Health (SMARTER) project developed the Sensor Common Metadata Specifications, a logical model organized into three domains: Instrument, Deployment, and Output.^11^^,^^17^^,^^18^ The Instrument domain captures physical and functional attributes, including capabilities and validation; the Deployment domain describes usage context, including calibration procedures; and the Output domain specifies measured entities and associated metadata. Collectively, these domains provide a comprehensive framework for describing sensor technologies. The SMARTER model is being designed for integration into informatics platforms such as the Exposure Health Informatics Ecosystem (EHIE)^19^ to promote interoperability and reuse. In this study, we specifically focus on the “Instrument” domain, and within that, the “Instrument” entity. This entity represents the core metadata about the physical sensor itself, including details necessary to identify, classify, and interpret its use in exposure health contexts. The associated attributes describe technical specifications, operational characteristics, and contextual deployment details. Currently, researchers must parse sensor documentation, including technical datasheets, manufacturer manuals, exposure health literature, and unstructured web content, to identify relevant sensor metadata. Populating SMARTER schema from heterogeneous, unstructured documentation also remains largely manual and unscalable. As the exposome increasingly relies on diverse sensor technologies, scalable and automated approaches to metadata extraction are essential to support efficient data integration into research workflows.^20^ Natural Language Processing (NLP) offers a promising solution to the challenges associated with extracting sensor metadata from unstructured and heterogeneous textual sources. By leveraging techniques such as named entity recognition, relation extraction, and language modeling,^21-23^ NLP systems can automatically identify, structure, and harmonize relevant metadata fields embedded in sensor documentation. Recent advancements in large language models (LLMs) greatly enhance this capability by enabling accurate understanding of complex domain-specific language, even in the absence of rigid templates or standard formats^24^^,^^25^ or costly training on specifically curated repositories.

NLP, when implemented with LLMs, has shown considerable promise for automated metadata extraction across diverse domains. For instance, prior work has demonstrated the capability of LLMs to extract metadata from scholarly research articles, aiming to identify references to datasets and associated metadata descriptors.^26^ Digital built environment has leveraged transformer-based language models and custom tokenizers to assign semantic tags to building sensor metadata, demonstrating over 70% tagging accuracy in real-world scenarios.^12^^,^^27^

Despite these advancements, there is currently no systematic or automated framework dedicated to the extraction of metadata about sensors themselves, in the context of exposure health, from diverse documents describing sensors and sensor deployments. This gap represents a significant opportunity for applying LLM-based NLP methods to automate the extraction and harmonization of human exposure sensor metadata across diverse sources. Our work addresses this gap by automating the extraction and harmonization of instrument-level metadata to populate the SMARTER schema and integrate it into the overarching EHIE architecture.^19^ The purpose of this study is to develop and evaluate an LLM-based approach for automatically extracting and harmonizing sensor metadata from unstructured exposure health literature. Specifically, we built and assessed a prototypical zero-shot extraction pipeline using GPT-4, iteratively refining prompts and applying NLP post-processing to align outputs with the SMARTER metadata schema.^11^^,^^17^ We adopted a zero-shot strategy to avoid the substantial cost and time required to curate a task-specific labeled corpus across heterogeneous sources, a process known to be a major bottleneck that typically demands domain experts for annotation.^28^ This automated framework addresses a critical bottleneck in environmental health research by significantly reducing the manual burden of metadata curation, improving the quality and consistency of extracted information, and enhancing the scalability of metadata repository development.

## Methods

**Figure 1.**
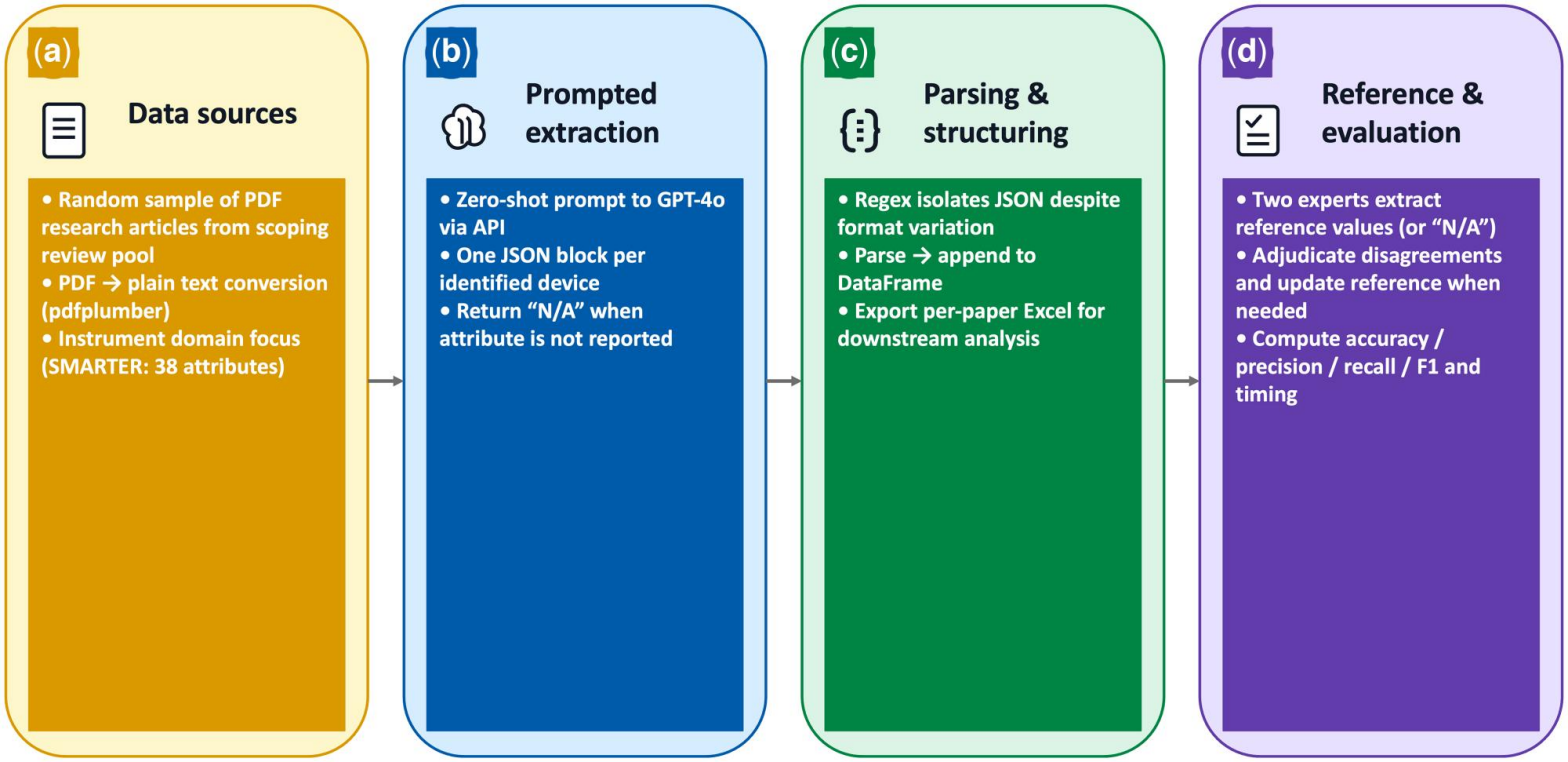
LLM-based sensor metadata extraction workflow. (a) Data source & selection: Twenty PDF research articles were randomly sampled from a prior exposure-health sensor scoping review spanning heterogeneous sensing technologies. (b) Data preprocessing: PDFs were converted to plain text (pdfplumber) to create LLM-ready inputs. (c) LLM extraction & structuring: GPT-4o (zero-shot; accessed November 5, 2025) extracted 38 SMARTER “Instrument” attributes per explicitly described device and returned a JSON-formatted response. (d) Parsing, aggregation & evaluation: Regular-expression–based JSON isolation and parsing produced per-paper structured outputs (Excel), which were compared against dual-expert reference standards with adjudication of disagreements and logging of processing time for efficiency analyses.

In this study we focus on the Instrument domain, specifically the Instrument entity which contains the core attributes needed to identify, classify, and interpret a sensor for exposure-health applications. The associated attributes describe technical specifications, operational characteristics, and contextual deployment details. The full list of attributes defined for this entity, along with descriptions can be found in the Supplementary Appendix A. These attributes include both required and optional fields, covering aspects such as model name, manufacturer, measured entities, power source, dimensions, usage context, and maintenance recommendations. Figure 1 summarizes the end-to-end workflow used in this study from PDF selection and preprocessing through LLM-based metadata extraction, JSON parsing/aggregation, and expert adjudication for evaluation.

### Data source

While diverse resources document sensor metadata, such as websites, datasheets, and catalogs, we focused on extracting the metadata embedded in PDF format from published research articles. Twenty papers were randomly selected from a pool of exposure health sensor studies identified through a prior scoping review of exposure health research studies. The original scoping review focused on identifying and screening studies involving environmental sensors across a wide range of exposure health domains. Notably, the scoping review included studies that used heterogeneous sensing technologies to measure a broad range of entities, including air pollutants, environmental conditions, physiological signals, geolocation data, and wireless communication indicators. The papers selected for evaluation in this study were randomly drawn from this diverse set of articles in order to support the framework’s relevance and adaptability across multiple exposure health contexts. This phase served as a foundational step for evaluating the feasibility and accuracy of LLM-based sensor metadata extraction.

In this study, we used the base version of the GPT-4o model for generative question answering. This model was accessed via OpenAI’s API in zero-shot learning setting. In the zero-shot learning setting, a model is presented with tasks or queries for which it has not received explicit training. It is expected to extrapolate knowledge from its pre-existing understanding of language and context to generate meaningful responses. This setting challenges the model to generalize effectively and showcase adaptability to novel prompts, reflecting its capacity to comprehend and manipulate language beyond the scope of its training data.^29^^,^^30^ Since prompt engineering is essential when interacting with any LLM to obtain high-quality responses,^31^ we first experimented with and formulated prompts to elicit the desired responses from the model. We then applied the finalized zero-shot prompt to extract sensor metadata, structured as 38 predefined attributes aligned with SMARTER metadata schema for each identified device. Our finalized prompt was selected to be as follows:

“Task Overview:

Given the extracted text from a research paper, identify and extract metadata related to every sensor device used in this study. The extracted information should be categorized into predefined entity labels. Ensure that the information is extracted accurately and presented in a structured JSON format:

Entity Labels and Their Definitions:

model_name:The term by which the instrument is known. This could be a trade name or an alias.

model_id: The unique identifier used to differentiate each model of an instrument made by certain manufacturer.

version_number: The current version of the instrument model. It differentiates instruments within the same model. They usually refer to a version of the hardware.

mobility: Whether the instrument can be moved around for measuring the species, A boolean indication (Yes/No).

measured_entities: List of all the types of measurement done by the instrument

firmware_software_version: Current firmware or software version of the model.

instrument_type: The category of instrument based on the species measured by the instrument.

manufacturer: The person, group, or organization that develops or produces the instrument.

patent_number: The serial number of the patent, if the instrument is patented.

patent_issued_country: The country issuing the patent.

dimensions: The size of the instrument in physical space. Use this if the dimensions aren’t available discretely, else use the below fields.

dimension_depth: The depth or thickness of the instrument.

dimension_height: The vertical height of the instrument.

dimension_length: The horizontal length of the instrument.

composition:The description of the composition of combining parts or elements making up of the instrument.

price:The cost of the instrument. This could be a potential price or price range of the instrument, such as the manufacturer recommended price, actual price, or price range to purchase the instrument.

price_type: Whether the price/price range is the potential price or actual price to purchase the instrument.

indoor_outdoor_use: Whether the instrument is intended to be used inside a building or structure that is protected from the natural environment. Or, if the instrument can be used outdoors and can tolerate exposure to the natural environment.

is_personal_device: Whether or not the instrument is intended to be used to and track information for individuals.

is_wearable_device: Whether or not the instrument can be worn by individuals on their body or carried, and track information. A boolean indication (Yes/No)

is_water_or_splash_proof: Whether or not the instrument can tolerate exposure to water. A boolean indication (Yes/No).

needs_power_source:Whether or not the instrument needs a source of power for its normal function. If power is needed, the type of power should be listed. See "Source of Power."

power_source: The type of power that supports the instrument for its normal function/s.

battery_operation_time_limit: The duration of battery life, if the "source of power" is battery.

battery_capacity: The amount of electric charge the battery can deliver at the rated voltage.

output_voltage: The voltage released by the battery.

is_rechargeable: Whether or not the battery’s electric charge can be restored by connecting the battery to a recharging device. A boolean indication (Yes/No).

battery_type: The category of battery, based on the chemical used in the battery’s electrochemical cells.

Charger: If the battery is rechargeable, this element is used to describe the charger.

time_to_full_charge: The time taken to recharge the battery.

has_display: Whether or not the instrument is capable of displaying information. If yes, more information can be recorded in the following data element, such as how many monitors, and what type of monitors does it have.

number_of_displays: The number of displays with the instrument.

display_type:The category of the monitor used to display information.

warranty_time: The length of time covered by the instrument’s warranty.

warranty_condition: The facts or conditions under which the warranty is valid.

lifetime_of_device: The duration of time during which the instrument is expected to function properly according to the manufacturer.

recommended_maintenance_method: The method suggested for maintaining the instrument.

recommended_maintenance_frequency: The frequency at which the maintenance should be repeated.

Instructions:

 • Extract relevant metadata from the provided text file, ensuring accuracy in categorization.
 • If information is missing, return “N/A” for that field.
 • Output the extracted metadata in a JSON format. Do not include additional information. I only need the JSON structured metadata for the sensors mentioned in this paper: < text here>.”

**Figure 2.**
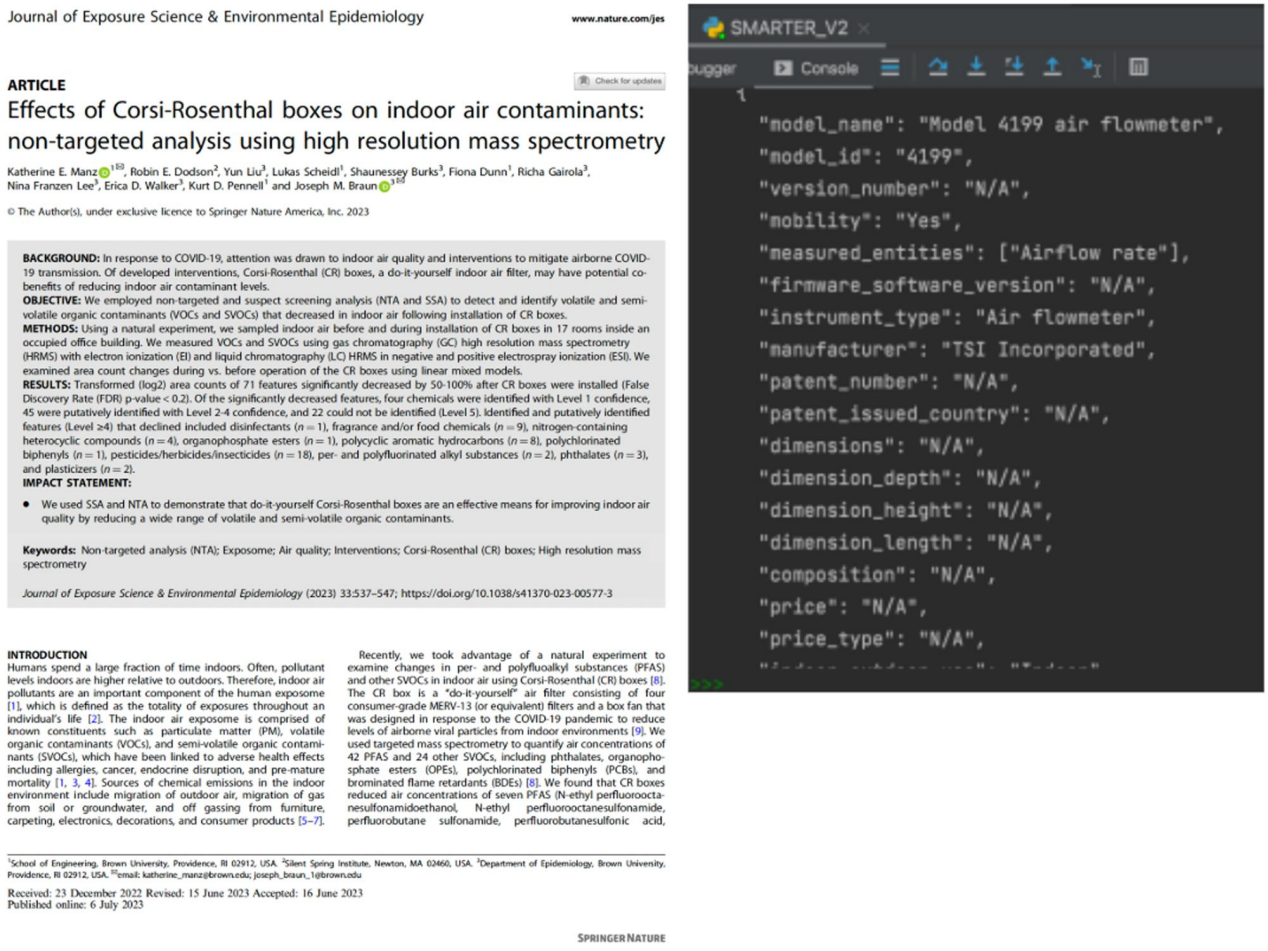
Example of input to GPT-augmented search (published journal article^32^) and output (sensor metadata in JSON format).

As a first step, we employed “*pdfplumber*” a Python library to convert each PDF document into a plain text file to ensure that the content could be effectively processed. These text files were then passed to the GPT-4o model based on the developed prompt. The model’s responses, which contained the extracted sensor metadata, were programmatically captured and saved in Excel format (Figure 2). Despite explicitly instructing the model to return output in strict JSON format, the actual responses from GPT-4o exhibited notable variation across documents. These inconsistencies included extra quotation marks, commas, or introductory phrases such as “the first sensor metadata” preceding the structured content. As a result, the format of GPT’s output was not uniform, making automated parsing and extraction of metadata challenging. To address this, we performed a manual review of all 20 outputs (already saved in an Excel file) to identify a generalized pattern capable of isolating the JSON-formatted metadata from surrounding text. We refined a regular expression pattern that could robustly extract JSON blocks from all papers regardless of their different formatting and validated this pattern on a new (previously unused) paper to ensure its generalizability. Each block is then parsed and appended to a growing Python DataFrame. Once parsing is complete, the full set of extracted metadata is saved as an Excel file, with one file generated per paper for downstream analysis.

### Reference standard preparation

Following the established methods for assessing agreement,^33^ two human experts independently performed metadata extraction by manually reviewing the same 20 research articles and extracted the same sensor metadata attributes for all sensors reported in the papers. The experts created a reference (the actual value when the attribute was reported, or “N/A” when it was absent). For each attribute, our pipeline returned either a textual value or the literal string “N/A” when the paper provided no information. Both experts compared GPT’s output to the reference and labeled it agree when (i) the attribute was present and the returned value matched the reference, or (ii) the attribute was absent and GPT correctly returned “N/A.” All other cases were labeled ‘not agree’ (eg, a wrong value for a present attribute, or a hallucinated value when the attribute was absent) along with an explanation.

For each sensor we summarized these binary judgments across all attributes to quantify the performance of the GPT model against human expert annotations by computing four standard evaluation metrics: precision, recall, accuracy, and F1-score. True positives were agree cases where an attribute was present and the value was correct; false negatives were not agree cases where an attribute was present but GPT returned an incorrect value or “N/A”; false positives were not agree cases where an attribute was absent but GPT returned a value; and true negatives were agree cases where an attribute was absent and GPT returned “N/A.” These metrics were calculated independently for each sensor and then aggregated to assess overall model effectiveness. Disagreements between the two experts were adjudicated through a structured consensus process. After completing independent reviews, the experts compared their agreement/non-agreement labels and accompanying rationales attribute-by-attribute. Any discordant judgments were flagged and jointly re-evaluated by returning to the source text to determine whether the attribute was truly reported and, if so, what the correct value should be. The final adjudicated label (agree Versus not agree) and, when applicable, the corrected reference value were then recorded as the consensus decision. As an example of adjudicated disagreement, one paper was assigned a reference value for the *model_name* attribute indicating the use of air-quality monitoring stations (“Not specified; fixed-site monitors operated by the Shanghai Environmental Monitoring Center (SEMC)”). This assignment was based on the statement: “Daily (24 h) mean air pollution concentration data, including PM2.5, PM10, NO2, SO2, and CO, from January 1, 2013 to December 31, 2014 were extracted from the Shanghai Environmental Monitoring Center (SEMC) database.” The LLM returned “N/A” (no sensing device recorded). One reviewer marked the LLM output as not-*agree* because the paper clearly relied on SEMC’s fixed-site monitoring network, even though no specific instrument model was named. The second reviewer marked it as *agree*, arguing that the text references a data repository rather than explicitly describing a sensor device and therefore does not provide sufficient evidence to populate the attribute “*model_name”*. The LLM returned value reflects a stricter interpretation that a sensor should be counted only when the article explicitly reports a device or monitoring instrument rather than only a data source. Both experts agreed the paper did not explicitly describe a sensing device (only a monitoring database/network), so the LLM output was accepted, and the reference value was updated. Moreover, there were a few cases where the LLM output reflected a plausible inference and the two experts initially disagreed on whether the attribute should be considered present or absent; after adjudication, the LLM-inferred value was accepted, and the reference label was updated accordingly.

Along with the duration of the human review, we recorded the processing time required for each paper, from the initial PDF-to-text conversion, through GPT-based metadata extraction, to save the final structured metadata in Excel format. These timestamps were systematically logged to enable direct comparison with the time taken by human experts to manually extract metadata from the same set of documents.

## Results

Through the manual review, a total of 36 sensors were identified across 20 research papers. Of these 20 papers, one did not explicitly report any sensor device, and two additional papers were ultimately labeled as containing no sensing device after expert disagreement was adjudicated, consistent with the LLM outputs, which had already labeled these papers as not recording any sensing device.

Table 1 presents the results of this manual extraction, showing how frequently each metadata attribute related to the “Instrument” entity was actually reported in the articles. Each row corresponds to the number of sensors (out of 36) for which that attribute was found and could be extracted manually (Coverage n), and the percentage of total sensors with that attribute reported (Coverage %). The attribute “measured_entities” was reported for 100% of sensors, while attributes “model_name” (83.33%), “mobility” (80.56%), “indoor_outdoor_use” (77.78%), “instrument_type” (75%), “is_personal_device” (75%), “is_wearable_device” (72.22%), and “manufacturer” (61.11%) were also commonly reported. In contrast, many technical specifications, such as “version_number”, “battery_capacity”, “output_voltage”, “charger”, and “warranty_time”, had 0% coverage, indicating they were not reported in any of the articles reviewed. This uneven distribution highlights that while commonly used fields are often reported, more detailed technical or maintenance-related information is rarely documented in research papers. To enrich the metadata landscape and address these reporting gaps, one promising direction is to supplement extraction from primary texts with retrieval of referenced manufacturer catalogs or technical documentation. Specifically, when a sensing device is mentioned in a paper, often by model name or manufacturer, it may be linked to external sources such as user manuals, datasheets, or product websites. Automatically identifying these references and retrieving the corresponding documents would allow for secondary metadata extraction from authoritative sources. Incorporating this complementary metadata, particularly for technical specifications, power requirements, and maintenance instructions, could significantly improve coverage and completeness of the “Instrument” entity in metadata schemas and enhance the robustness of downstream analyses in exposome and environmental health research.

**Table 1.** Coverage of papers for attributes for the “instrument” entity.

Attribute	Coverage (n)	Coverage (%)
Model_name	30	83.33
Model_id	12	33.33
Version_number	0	0
Mobility	29	80.56
Measured_entities	36	100
Firmware_software_version	1	2.78
Instrument_type	27	75
Manufacturer	22	61.11
Patent_number	0	0
Patent_issued_country	0	0
Dimensions	2	5.56
Dimension_depth	2	5.56
Dimension_height	2	5.56
Dimension_length	2	5.56
Composition	10	27.78
Price	1	2.78
Price_type	0	0
Indoor_outdoor_use	28	77.78
Is_personal_device	27	75
Is_wearable_device	26	72.22
Is_water_or_splash_proof	2	5.56
Needs_power_source	13	36.11
Power_source	6	16.67
Battery_operation_time_limit	2	5.56
Battery_capacity	0	0
Output_voltage	0	0
Is_rechargeable	2	5.56
Battery_type	0	0
Charger	0	0
Time_to_full_charge	0	0
Has_display	4	11.11
Number_of_displays	1	2.78
Display_type	0	0
Warranty_time	0	0
Warranty_condition	0	0
Lifetime_of_device	0	0
Recommended_maintenance_method	1	2.78
Recommended_maintenance_frequency	1	2.78

Table 2 presents the performance of GPT in the zero-shot setting, accessed on November 5, 2025, and evaluated against the expert-adjudicated reference labels across the 36 identified sensors and their corresponding 38 metadata attributes in terms of accuracy, precision, recall, and F1-score. Metrics were calculated per paper and per sensor across all attributes. Across most papers/sensors, performance is consistently high (accuracy ≈ 0.89-1.00, F1 ≈ 0.94-1.00), and recall is 1.00 for nearly all non-zero rows, indicating that, when GPT identifies a sensor and produces an output, it generally captures the attributes that were present in the reference. The rows with all zeros represent a different failure mode: GPT did not extract those sensors at all, so no attribute-level matches were possible, and the resulting metrics collapse to zero. These misses cluster notably in Paper 5 (sensors #3–#8) and Paper 16 (sensor #1), suggesting that the dominant source of error is not attribute parsing once a sensor is found, but rather the sensor-identification step.

**Table 2.** Performance metrics per sensor.

Paper #	Sensor #	Accuracy	Precision	Recall	F1_score	Hallucination rate (%)
1	1	1	1	1	1	0
	2	0.97	0.97	1	0.99	0
2	1	0.95	0.95	1	0.97	2.63
	2	0.92	0.92	1	0.96	2.63
3	1	0.92	0.92	1	0.96	2.63
	2	0.95	0.95	1	0.97	0
4	1	0.95	0.95	1	0.97	0
5	1	0.89	0.89	1	0.94	7.90
	2	0.92	0.92	1	0.96	5.26
	3	0	0	0	0	N/A
	4	0	0	0	0	N/A
	5	0	0	0	0	N/A
	6	0	0	0	0	N/A
	7	0	0	0	0	N/A
	8	0	0	0	0	N/A
6	1	0.97	0.97	1	0.99	0
7	1	0.95	0.95	1	0.97	5.26
8	1	0.97	0.97	1	0.99	0
9	1	0.95	0.95	1	0.97	5.26
10	1	0.97	0.97	1	0.99	0
	2	0.97	0.97	1	0.99	0
11	1	1	1	1	1	0
12	1	0.97	0.97	1	0.99	10.53
	2	0.97	0.97	1	0.99	10.53
	3	0.97	0.97	1	0.99	10.53
	4	0.97	0.97	1	0.99	10.53
13	1	0.92	0.92	1	0.96	5.26
	2	0.95	0.95	1	0.97	2.63
14	1	0.95	0.95	1	0.97	5.26
	2	0.95	0.95	1	0.97	5.26
15	1	0.89	0.89	1	0.94	0
16	1	0	0	0	0	N/A
	2	0.89	0.89	1	0.94	0
17	1	0.89	0.89	1	0.94	5.26
	2	0.92	0.92	1	0.96	2.63
	3	0.95	0.95	1	0.97	2.63

Table 2 also records the hallucination rate per sensor, computed as the number of attributes where the LLM produced a value despite the reference being “N/A,” divided by the total number of attributes (ie, 38). Note that an alternative definition for hallucination rate is to divide by the number of attributes labeled “N/A” in the reference for that sensor. Still, because the count of “N/A” attributes varies across sensors (ie, different denominators per device), we report the hallucination rate using a fixed denominator (38 attributes) to ensure comparability across sensors and papers. In our evaluation, a hallucination is defined as any instance where an attribute’s reference value was “N/A” (ie, the paper did not report that attribute), but the LLM nonetheless assigned a non-empty value for that attribute. Notably, there were a few cases where the LLM output reflected a plausible inference and the two experts initially disagreed on whether the attribute should be considered present; after adjudication, the LLM-inferred value was accepted, and the reference label was updated accordingly (these adjudicated updates were not counted as hallucinations under our definition). Across the sensors for which a hallucination rate could be computed (29/36), hallucinations were generally infrequent: the median hallucination rate was 2.63% (≈ 1 hallucinated attribute out of 38), and the mean was 3.54%. Eleven sensors had 0% hallucination, while the remaining sensors most commonly showed 2.63% (6 sensors; 1 attribute) or 5.26% (7 sensors; 2 attributes), with only a small subset reaching higher rates (7.90% for 1 sensor; 3 attributes, and 10.53% for 4 sensors; 4 attributes), indicating that when hallucinations occurred they were typically limited to a few fields rather than widespread fabrication across the metadata profile.

Table 3 summarizes the average per-paper accuracy, precision, recall, and F1 for GPT’s extraction, and the final row reports macro-averages: the average of each metric across the 17 papers. Across the 17 papers, the model achieved consistently high average performance in most studies, with average accuracy/precision typically ∼0.92-1.00 and average recall = 1.00 for the majority of papers, yielding average F1 scores around 0.96-1.00. This pattern indicates that when sensors were clearly described as being used in the study, the LLM reliably extracted the required metadata and rarely missed attributes that were actually reported.

**Table 3.** Performance metrics per paper and overall performance.

Paper #	Average accuracy	Average precision	Average recall	Average F1 score
1	0.98	0.98	1	1
2	0.94	0.94	1	0.96
3	0.94	0.94	1	0.96
4	0.95	0.95	1	0.97
5	0.23	0.23	0.25	0.24
6	0.97	0.97	1	0.99
7	0.95	0.95	1	0.97
8	0.97	0.97	1	0.99
9	0.95	0.95	1	0.97
10	0.97	0.97	1	0.99
11	1	1	1	1
12	0.97	0.97	1	0.99
13	0.94	0.94	1	0.96
14	0.95	0.95	1	0.97
15	0.89	0.89	1	0.94
16	0.44	0.44	0.5	0.47
17	0.92	0.92	1	0.96
Overall	0.88	0.88	0.93	0.90

The overall metrics (accuracy = 0.88, precision = 0.88, recall = 0.93, F1 = 0.90) are lower mainly because performance was dominated by a small number of papers, especially Paper 5 (F1 = 0.24) and Paper 16 (F1 = 0.47). These low averages are consistent with error-analysis findings: these papers included multiple sensor mentions where several devices were not extracted at all (often because they appeared as comparator/reference instruments or in literature-context statements rather than being explicitly deployed in the current study). As a result, recall dropped in those papers, pulling down the overall recall and, consequently, the overall F1 despite strong performance elsewhere.

The comparison of extraction time between manual and automated methods reveals a substantial efficiency gain achieved through automation. On average, manual extraction of sensor metadata from each paper took approximately 2,380s (about 39min), while the automated pipeline completed the same task in approximately 21s. This results in a mean time saving of 2,359s per document, translating to a 99% reduction in execution time. Overall, the automated method was approximately 174 times faster than the manual process.

## Discussion

The GPT demonstrated the ability to infer metadata values that were not explicitly stated in the text. In multiple cases, the model successfully filled in missing details through contextual reasoning. All such inferred values were verified to be correct by expert reviewers. This suggests that GPT can effectively generalize from surrounding content to enhance metadata completeness. In some cases, GPT also extracted relevant non-sensor devices such as air pumps as distinct devices. Although these instruments do not meet strict definitions of a sensor, they are integral components of data acquisition and collection workflows and can materially influence the quality of the resulting data.

Most articles in the corpus were primarily focused on the application of sensor devices in exposure health studies rather than on the design or engineering specifications of the sensors themselves. As a result, metadata was often sparse or incomplete, both for automated and manual extraction methods. Table 1 highlights this issue. While some basic descriptive metadata was consistently available, more granular or engineering-specific metadata was often omitted. Continuing the issue of metadata sparsity, many articles referenced external sources for sensor specifications rather than describing them directly. This practice, along with the common placement of sensor details in brief “Methods” sections, often limited the extractable information about the sensors. One article described downstream analysis using chromatography, but did not provide details on how the air samples were originally collected, instead referencing an external publication. Future work should refine the prompt, clarifying the distinction between primary study sensors and supporting/reference devices, and, where helpful, using a small set of few-shot examples with added reasoning guidance, to extract metadata for both primary and explicitly named benchmark instruments while still excluding mentions that only refer to monitoring networks, datasets, or organizations without describing a specific device. Future extractions could also benefit from citation traversal to retrieve linked sampling metadata.

Overall, the GPT-based pipeline substantially outperformed manual methods in speed of metadata extraction. It demonstrated high precision, recall, and overall performance, highlighting the strong potential GPT to streamline and scale sensor metadata extraction with minimal loss in quality. Despite its strong potential, the pipeline also revealed the need for a more adaptive and agentic approach to metadata extraction. Since metadata is often dispersed across multiple sources, an effective extraction framework must be capable of traversing citations and external references, reconciling conflicting metadata values across documents and against existing records in a metadata repository and querying the internet for supplemental information. Moreover, the time-saving ratio recorded in this study reflects only per-document extraction runtime (human abstraction time versus LLM inference and post-processing time) and does not include the fixed, upfront development costs required to operationalize the automated pipeline. In practice, substantial one-time effort was needed to implement the extraction workflow (eg, code development, prompt refinement, and rule-based regular-expression design), and these setup activities can be non-trivial for a new project or a new metadata schema. Accordingly, the efficiency gains reported here should be interpreted as benefits that accrue with scale: once the pipeline is established, the marginal cost of processing additional papers becomes very small, making automation increasingly advantageous as the number of documents grows. Additionally, the pipeline should integrate human-in-the-loop mechanisms to support adjudication and expert curation, as well as track versioning and updates to stored metadata values over time.

This study demonstrates the use of AI methods to make sensors and, in turn, the exposome FAIR (Findable, Accessible, Interoperable, and Reusable).^34^^,^^35^ By reducing the effort required to discover and harmonize sensor metadata, this FAIRification of sensors provides means to easily select, obtain, deploy, and reuse sensors across exposure health, and provide a metadata-enrichened approach to construct exposomes by assimilating exposure profiles from prospective sensor deployments as well as historic, ambient, and other real-world measurements.

## Conclusion and future work

This study presents a novel application of LLMs, specifically GPT-4, to automate the extraction of sensor metadata from unstructured exposure health literature. By focusing on the “Instrument” entity within the SMARTER metadata model, we demonstrated that GPT can significantly reduce the time and labor required for metadata curation, achieving extraction speeds 174 times faster than manual review and maintaining high performance across key evaluation metrics. Our results confirm the feasibility of using LLMs for structured metadata extraction in domains where sensor documentation is often sparse, heterogeneous, and unstandardized. Moreover, the GPT showed strong contextual reasoning, successfully inferring missing values, but occasionally misclassified supporting instruments as sensors. Future work should refine the prompt, clarifying the distinction between primary study sensors and supporting/reference devices and, where helpful, using a small set of few-shot examples with added reasoning guidance, to extract metadata for both primary and explicitly named benchmark instruments while still excluding mentions that only refer to monitoring networks, datasets, or organizations without describing a specific device. Our findings also underscore the need to develop more adaptive pipelines that incorporate citation traversal, metadata repository integration, and human-in-the-loop curation, while also mitigating erroneous attribute filling, as reflected in the observed hallucination rates. Enhancing these capabilities will enable scalable, accurate, and continuously updated metadata infrastructure to support sensor integration in exposure health research.

## Supplementary Material

osag008_Supplementary_Data

## Data Availability

The data underlying this article are available at: https://zenodo.org/records/18500477.
